# Improving Normal/Abnormal and Benign/Malignant Classifications in Mammography with ROI-Stratified Deep Learning

**DOI:** 10.3390/bioengineering13020206

**Published:** 2026-02-12

**Authors:** Kenji Yoshitsugu, Kazumasa Kishimoto, Tadamasa Takemura

**Affiliations:** 1 Graduate School of Information Science, University of Hyogo, 7-1-28 Minatojima Minamimachi, Chuo-ku, Kobe-shi 650-0047, Hyogo, Japan; takemura@ai.u-hyogo.ac.jp; 2 Division of Medical Information Technology and Administration Planning, Kyoto University Hospital, 54 Kawaramachi, Shogoin, Sakyo-ku, Kyoto 606-8507, Japan; kishimoto@kuhp.kyoto-u.ac.jp

**Keywords:** deep learning, mammography, region of interest, ROI-stratified

## Abstract

Deep Learning (DL) has undergone widespread adoption for medical image analysis and diagnosis. Numerous studies have explored mammographic image analysis for breast cancer screening. For this study, we assessed the hypothesis that stratifying mammography images based on the presence or absence of a corresponding region of interest (ROI) improves classification accuracy for both normal–abnormal and benign–malignant classifications. Our methodology involves independently training models and performing predictions on each subgroup with subsequent integration of the results. We used several DL models, including ResNet, EfficientNet, SwinTransformer, ConvNeXt, and MobileNet. For experimentation, we used the publicly available VinDr., CDD-CESM, and DMID datasets. Our comparison with prediction results obtained without ROI-based stratification demonstrated that the utility of considering ROI presence to enhance diagnostic accuracy in mammography increases along with the data volume. These findings support the usefulness of our stratification approach, particularly as a dataset’s size grows.

## 1. Introduction

### 1.1. Global Burden of Breast Cancer: Current Status and Future Projections

Breast cancer poses an important threat to women’s health worldwide. Its burdens vary considerably across regions and socioeconomic conditions. This section provides an overview of the current global incidence and mortality rates of breast cancer based on GLOBOCAN 2022 data [1], with discussion of regional disparities and their underlying factors, in addition to future projections.

According to GLOBOCAN 2022 data [1], approximately 2.3 million new breast cancer cases occurred globally in 2022, causing approximately 670,000 deaths. The age-standardized incidence rate (ASR) was 46.8 per 100,000. The age-standardized mortality rate (ASR) was 12.7 per 100,000. These figures demonstrate clearly that breast cancer persists as a crucially important public health issue.

Breast cancer incidence and mortality rates differ considerably among regions, exhibiting particular dependence on the Human Development Index (HDI). High-HDI regions (e.g., Europe, North America, Oceania) tend to have higher incidence rates, whereas low-HDI regions (e.g., Africa) are associated with higher mortality rates. These disparities are likely to stem from differences in screening availability, stage at diagnosis, treatment infrastructure, and socioeconomic factors.

A study by Liao et al. [2] indicated that breast cancer burdens are likely to increase in the future. Specifically in Africa, new cases among young women (0–39 years) are projected to increase by approximately 96%: from 43,168 cases in 2022 to 84,683 cases in 2050. The Asian region has the greatest number of cases. The total number is projected to remain high, but with variations depending on the HDI level.

The economic burdens of breast cancer vary considerably among nations and regions. A study particularly addressing India [2] projected the numbers of breast cancer patients and associated economic burdens during 2021–2030. The study found that the total economic burden would increase from approximately US$8 billion in 2021 to approximately US$13.95 billion in 2030. Additionally, a study of chemotherapy patients in an Ethiopian hospital [3] showed that the average total direct cost for outpatients was approximately US $1188.26, suggesting heavy patient burdens in low-income and middle-income countries.

Breast cancer presents severe global health difficulties, with large regional disparities. To reduce future breast cancer burdens, it is crucially important to improve screening coverage, early diagnosis, and access to appropriate treatment. High mortality rates in low-HDI regions are a particularly urgent issue. International cooperation and support are fundamentally important.

### 1.2. Utility of Deep Learning-Based Mammography Imaging Diagnosis in Breast Cancer Treatment Planning

Deep learning has emerged as a transformative approach used for medical imaging diagnosis, particularly for cancer detection. Yuliana Jiménez-Gaona et al. [4] presented a comprehensive review of deep learning applications using ultrasound and mammography for breast cancer diagnosis, analyzing 59 studies from 250 research articles published during 2010–2020. Results show that deep learning-based computer-aided diagnosis systems reduce manual feature extraction needs effectively and improve diagnostic accuracy. Munir et al. provided a bibliographic review covering various deep learning techniques including Convolutional Neural Networks (CNNs), GANs, and RNNs for cancer diagnosis across breast, lung, brain, and skin cancers, highlighting the superiority of AI methods over traditional diagnostic approaches [5]. By contrast, Nagendran et al. identified important methodological concerns related to deep learning studies, finding that only 9 of 81 non-randomized trials were prospective, with high risk of bias in 58 studies and limited data availability [6]. Kassem et al. reviewed 102 reports of studies of skin lesion diagnosis, comparing traditional machine learning with deep learning methods while identifying challenges including small datasets and evaluation biases [7].

### 1.3. Deep Learning-Based Methods for Mammographic Image Diagnosis

Since the advent of deep learning technologies, particularly Convolutional Neural Networks, research in breast cancer detection and diagnosis from mammograms has advanced dramatically. The approaches adopted for these studies fall into two main streams based on the granularity of the annotation information they use and the format of the model’s input. These are region of interest (ROI)-based methods, which specifically examine candidate lesion areas, and whole-image-based methods, which analyze the entire image directly. This report outlines these two primary methodology categories and presents key publications in chronological order to demonstrate the development and diversity within each stream.

#### 1.3.1. RO-Based Methods

ROI-based methods feature a two-stage approach. The first stage detects a candidate lesion region within the image. The second stage inputs a cropped patch of that region into a classification model to perform a diagnostic assessment (e.g., benign or malignant). This method is well-suited to learning fine-grained morphological features because it allows the model to emphasize high-resolution information of the lesion for its processes. Nevertheless, this approach has an important limitation: its final diagnostic performance is strongly dependent on the accuracy of the ROI detection stage. Its training requires precise, local annotations that indicate the lesion location.

The following list presents chronologically ordered descriptions of major academic efforts in this method category.

Levy et al. presented a methodology that employs CNNs to classify pre-segmented breast masses on mammograms directly as benign or malignant. To overcome the challenge of limited training data, this approach used a combination of transfer learning, meticulous pre-processing, and data augmentation. Results show that the methodology achieved high classification accuracy on the DDSM dataset, surpassing human performance. It also demonstrated model interpretability [8].

Kooi et al. directly compared the performance of a conventional Computer-Aided Diagnosis (CAD) system based on hand-crafted features with a CNN using approximately 45,000 mammograms. The CNN outperformed the conventional CAD system at the low-sensitivity regime and performed comparably at the high-sensitivity regime. Furthermore, a patch-level reader study found no significant difference between the CNN performance and that of certified screening radiologists [9].

Shen et al. developed an end-to-end approach for deep learning-based mammography diagnosis. The method reduces reliance on expensive local annotations using lesion-level annotations only during the initial training stage and subsequent training on image-level labels. The approach demonstrated high performance when using the CBIS-DDSM (area under the curve, AUC 0.91) and INbreast (AUC 0.98) datasets [10].

Al-antari et al. proposed a deep learning-based CAD system that integrates breast mass detection (YOLO), segmentation (using the proposed FrCN method), and benign–malignant classification (CNN) into a single framework. In a four-fold cross-validation on the public INbreast dataset, the system achieved 98.96% detection accuracy, 92.97% segmentation accuracy, and 95.64% classification accuracy (AUC 94.78%), demonstrating its effectiveness over conventional methods [11].

Chougrad et al. developed a CAD system that classifies mammography ROIs as benign or malignant using CNNs and transfer learning. The research explored optimal fine-tuning strategies and used a merged dataset from multiple public sources for training. Upon validation on the independent MIAS dataset, the system achieved 98.23% accuracy and 0.99 AUC, demonstrating high generalization performance [12].

Sun et al. proposed a multi-view CNN to leverage complementary information from craniocaudal (CC) and mediolateral oblique (MLO) mammographic views. The methodology featured a penalty term to enforce inter-view consistency and the learning of invariant representations via Supervised Contrastive Learning (SCL). Through experiments conducted of the DDSM dataset, the study demonstrated superior classification performance and diagnostic speed compared to conventional methods [13].

Garrucho et al. proposed a single-source training pipeline that requires no images from unseen target domains. In an evaluation across five unseen domains, the proposed methodology outperformed conventional transfer learning approaches in four of the five domains and reduced the domain shift attributable to different acquisition systems. The study also analyzed the effects of covariates such as patient age and breast density [14].

Celat et al. evaluated the performance of a deep learning model (ResNet152V2) for classifying normal–abnormal mammography ROIs using the large-scale EMBED dataset, achieving an AUC of 0.975. Subgroup analysis revealed significant associations between false-negative predictions and white patients or architectural distortions, as well as significant associations between false-positive predictions and high breast density. This finding underscores the importance of subgroup analysis for improving model fairness and interpretability [15].

Camurdan, O et al. combined a patch-based deep learning approach with curriculum learning to produce a system for mammographic breast cancer detection. Even when using only limited strong annotations (20%) and weak annotations (image labels), the method improved both the F1 score and Grad-CAM-based explainability (Ground Truth overlap ratio) compared to a baseline trained with no strong annotation. This performance trend held also for an external dataset, demonstrating the effectiveness of this annotation-efficient model [16].

#### 1.3.2. Whole-Image-Based Methods

Whole-Image-based methods perform end-to-end diagnosis by feeding the entire mammogram directly into a model, consequently bypassing an earlier ROI detection step. This approach holds the advantage of requiring only image-level diagnostic labels for training, which reduces annotation costs significantly. However, because lesion areas are extremely small relative to the entire image, recent frameworks in weakly supervised learning and Multiple Instance Learning (MIL) are crucially important for overcoming this challenge.

The following list presents a chronologically ordered listing of major academic papers in this method category.

Campanella et al. proposed an MIL system that trains using only slide-level diagnoses, obviating the need for costly pixel-level annotations. In a large-scale evaluation using 44,732 whole slide images, the system achieved an AUC greater than 0.98 for all three cancer types. For clinical application, this system could potentially exclude 65–75% of slides while maintaining 100% sensitivity [17].

McKinney et al. developed an AI system for breast cancer screening. We evaluated the system using datasets from the UK and the US, for which it reduced the rates of false positives and false negatives significantly. In an independent study comparing the AI against six radiologists, the system outperformed all human readers, exceeding the average radiologist’s AUC by an absolute margin of 11.5%. Furthermore, in a double-reading simulation, the AI system maintained non-inferior performance while reducing the workload of the second reader by 88% [18].

Lotter et al. used a whole-image-based approach (including MIL) to demonstrate that a high-accuracy diagnostic model can be built even from limited annotations, thereby proving the effectiveness of annotation-efficient learning [19].

Zhu et al. proposed an end-to-end deep MIL network for whole mammogram classification, thereby eliminating the need for costly ROI annotations. The authors explored three MIL schemes. Through experimentation on the INbreast dataset, they demonstrated the robustness of their approach compared to conventional methods that require annotation [20].

El Mikaty et al. proposed FPN-MIL, a model integrating a Feature Pyramid Network (FPN), to address the challenge of multi-scale lesions that conventional MIL for mammography overlooks. The proposed method realizes multi-scale analysis from single-scale patch inputs and employs an attention-based aggregation mechanism to enhance robustness against scale variations. Experiment results demonstrated that the proposed method surpassed conventional approaches; FPN-SetTrans achieved the best performance for calcification classification, whereas FPN-AbMIL performed best for mass classification [21].

Deep learning for mammography diagnosis shows a clear shift in research focus from ROI-based methods, which precisely analyze local features, to whole-image-based methods, which reduce annotation costs and can capture global context. Particularly, the adoption of weakly supervised learning and MIL offers the potential to combine advantages of both approaches. It represents a primary direction for future research and development. Furthermore, the scope of applications is expanding from simple benign–malignant classification to risk and treatment response prediction, progressively increasing the clinical value of these systems.

### 1.4. Positioning and Objective of Our Study

The methodology proposed herein falls under the category of image classification. In earlier work [22], we introduced and evaluated an ROI-stratified approach by which we separate images based on the presence or absence of an annotated ROI for distinct training and inference pipelines before integrating their results. Using two public datasets and two deep learning models, we demonstrated that this method has potential to improve benign–malignant classification accuracy.

For this work, we assess the generalizability of our ROI-stratified approach by application of it to two distinct classification tasks (normal versus abnormal and benign versus malignant) across three public datasets and five representative deep learning models.

The innovation and uniqueness of this study lie in our methodology: after preprocessing the mammograms images, we partition the images based on the presence or absence of Regions of Interest (ROIs) for separate training and prediction, and subsequently conbine these prediction results.

## 2. Methods

### 2.1. Overview

**Figure 1:**
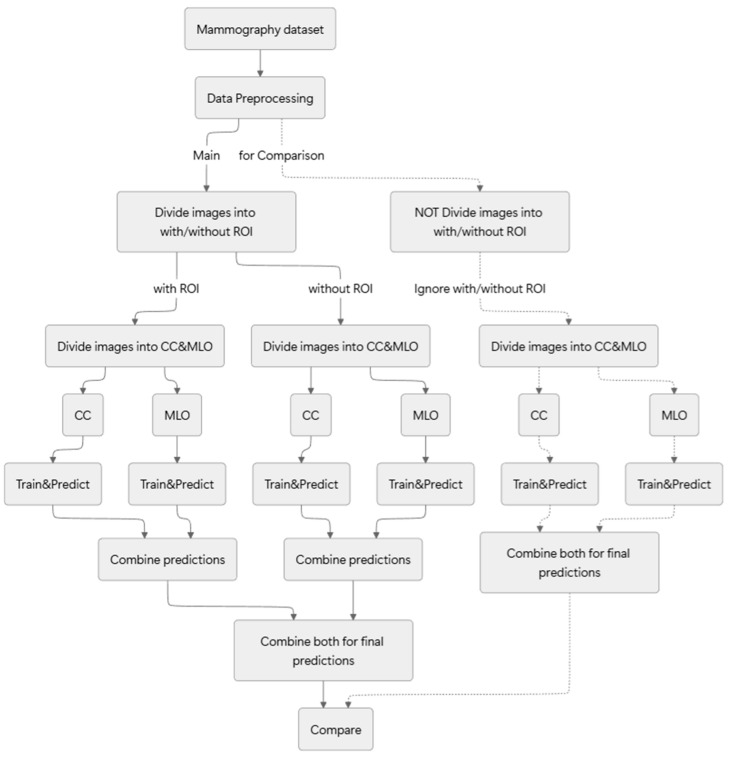
Overview of the proposed classification methodology.

Figure 1 portrays an overview of the method proposed and evaluated with this study.

For this study, we introduce a methodology that first stratifies the dataset based on two criteria: the presence or absence of a ROI and the mammographic view type (craniocaudal, CC; mediolateral oblique, MLO). After this process creates four distinct data streams, we perform separate training and inference on each stream and subsequently integrate the four resulting predictions. We apply this entire process in a two-stage cascade: an initial normal-versus-abnormal.

### 2.2. Preprocessing

**Figure 2:**
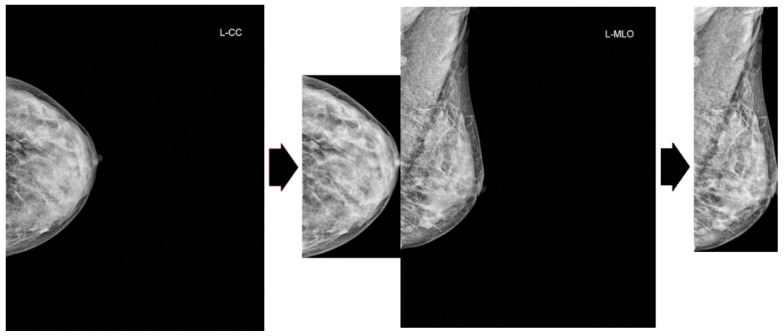
Cropping of excess black background. Original images are from the VinDr dataset.

Our preprocessing pipeline comprises two sequential steps. First, we apply Contrast Limited Adaptive Histogram Equalization (CLAHE) to mammogram images to enhance local contrast. Subsequently, we crop the excess black background from the images, as portrayed in Figure 2.

### 2.3. Deep Learning Models

We selected the five deep learning models used for this study to represent the major architectural paradigms in modern computer vision and to evaluate their technological progression systematically. First, we establish ResNet50 [23], an architecture that enabled the training of deep networks through its use of residual connections, as the objective baseline for all performance comparisons. To represent key evolutionary paths for CNNs, we selected EfficientNet-B4 [24], which has systematized the tradeoff between performance and efficiency via compound scaling, and MobileNetV3 [25], a leading lightweight model designed for computational efficiency. Their inclusion enables us to evaluate models along different optimization axes.

Furthermore, to represent the major recent trend of Transformers [26], we adopt Swin Transformer [27], which features a hierarchical structure and a windowed self-attention mechanism. As a direct counterpart, we selected ConvNeXt [28], a latest-generation CNN that reconstructs design principles of Swin Transformer within a purely convolutional framework. This overall configuration supports a multi-faceted comparative analysis of performance, efficiency, and fundamental computational principles, extending from established standard architectures to the latest design philosophies.

### 2.4. Hyperparameter Optimization

To maximize the generalization performance of each model, our hyperparameter optimization process used a stratified k-fold cross-validation framework. This approach mitigates risks of overfitting to a single validation set and enables reliable performance estimation on imbalanced data. After applying Random Search as the search strategy for its efficiency in high-dimensional spaces, we constrained the search space to key parameters with a high contribution to performance: learning rate, batch size, and weight decay. The evaluation metric for each parameter combination was the mean validation AUC across all folds. To ensure computational tractability, we concurrently used an early stopping mechanism to prune unpromising trials and used a caching system for previously computed results.

We set the input image resolution to 380 × 380 for the EfficientNet model and 224 × 224 for the remaining models, consistent with the default value for each respective architecture.

### 2.5. Addressing Class Imbalance

To address class imbalance difficulties inherent in the dataset used for this study, we applied a weighted random sampling strategy to the training data. To each training sample, this method assigns a weight that calculated as the inverse of its class frequency. The method then constructs mini-batches by sampling instances with a probability proportional to their assigned weights. This process balances the class distribution observed by the model during training, aiming at mitigation of overfitting to the majority class while improving the discriminative performance for the minority class. We applied this sampling strategy exclusively during the training phase. For validation and testing, we used the original data distribution to ensure unbiased measurement of the generalization performance.

### 2.6. Data Augmentation

For this study, we introduce data augmentation to improve the generalization performance and robustness of our deep learning models and to mitigate overfitting on the limited training dataset. For implementation, we adopt the Albumentations library, which was selected for its computational efficiency and for its comprehensive suite of transformation techniques.

For the training data, in addition to resizing images to each model’s required input dimensions, we stochastically apply a pipeline of geometric and photometric transformations online. This pipeline includes the following: horizontal flipping; minor affine transformations such as shifting, scaling, and rotation; random adjustments to brightness and contrast; and the addition of Gaussian noise. This process maximizes the diversity of the data presented to the model during training. By contrast, we apply only deterministic preprocessing (resizing and normalization) to the evaluation data (validation and test sets). This asymmetrical approach ensures that we present diverse data patterns to the model during training. This approach simultaneously guarantees a fair and consistent measurement of its generalization performance during evaluation.

### 2.7. Evaluation Metrics

We used sensitivity, specificity, F-score, and accuracy as the evaluation metrics. These four evaluation metrics are defined by Equations (1)–(4), where TP, FP, TN, and FN respectively denote True Positives, False Positives, True Negatives, and False Negatives.

Sensitivity = TP/(TP + FN)(1)

Specificity = TN/(TN + FP)(2)

F-score = 2(TP/(TP + FP) × Sensitivity))/(TP/(TP + FP) + Sensitivity)(3)

Accuracy = (TP + TN)/(TP + FP + TN + FN)(4)

### 2.8. Computational Environment

Experiments were conducted using an OS (Windows 11 Pro; Microsoft Corp (One Microsoft Way, Redmond, WA 98052-7329, USA)) running on a system equipped with a 3.00 GHz processor (13th Gen Core (TM) i9-13900K; Intel Corp (2200 Mission College Blvd. Santa Clara, CA 95054 USA)), 128 GB of memory, and GPU (RTX 3090; NVIDIA Corp (2788 San Tomas Expressway, Santa Clara, CA 95051, USA)).

## 3. Materials

This study uses the following three publicly available datasets. Few public datasets offer ROI annotations with accompanying three-class labels (Normal, Benign, Malignant).

### 3.1. VinDr

This study uses the large-scale, publicly available VinDr-Mammo dataset [29] for training and evaluation of deep learning models for mammogram analysis. The dataset comprises approximately 20,000 Full-Field Digital Mammography (FFDM) images corresponding to 5000 studies, collected from clinical practice at two institutions in Vietnam. Its key feature is the detailed set of annotations provided by experienced radiologists. Each image includes an American College of Radiology (ACR) BI-RADS category, with bounding boxes to localize abnormal regions.

This study uses these pathological diagnosis labels as the ground truth for our classification task. Furthermore, the dataset provides officially defined training and test splits. Our experiments adhere strictly to this official partition to ensure the reproducibility of our results and to maintain comparability with other studies that use the same dataset.

Table 1 and Table 2 present the data composition of this dataset. The VinDr dataset provides BI-RADS information, but it lacks explicit benign–malignant classifications. Consequently, images categorized as BI-RADS 2 and BI-RADS 3 were classified as benign lesions, whereas those categorized as BI-RADS 4 and BI-RADS 5 were classified as malignant. Abnormal data consists of benign data and malignant data.

### 3.2. CDD-DESM

For this study, we used Categorized Digital Database for Low energy and Subtracted Contrast Enhanced Spec-tral Mammography (CDD-CESM) [30] as a second publicly available dataset for training and evaluating our deep learning model for mammographic image analysis.

Full medical reports are also provided for each case (DOCX) along with manual segmentation annotation for the abnormal findings in each image (CSV file). Each image with its corresponding manual annotation (breast composition, mass shape, mass margin, mass density, architectural distortion, asymmetries, calcification type, calcification distribution, mass enhancement pattern, non-mass enhancement pattern, non-mass enhancement distribution, and overall BIRADS assessment) is compiled into a single Excel (Microsoft Corp.) file.

Table 3 and Table 4 present the data composition of this dataset. Because the original release of this dataset does not include a predefined partition, we created a custom split for our experiments, dividing the data into training and test sets at an approximate 9:1 ratio. Abnormal data consists of benign data and malignant data.

### 3.3. DMID

For this study, we used DMID [31] as a third publicly available dataset for training and evaluating our deep learning model for mammographic image analysis. The DMID dataset, which includes images of mammograms, can be used for research and education purposes only. The dataset includes DCM images, TIFF images, a Radiology report, a Segmented mask, pixel level annotation of abnormal regions, and a csv file that includes other metadata [31].

In this dataset, each ROI within an image has a corresponding pathological diagnosis. Consequently, a single image can be associated with multiple findings of varying severity (e.g., Normal, Benign, Malignant). To assign a definitive, image-level label in such cases, we apply a hierarchical rule that prioritizes the most severe diagnosis. We define the hierarchy as Malignant over Benign, and Benign over Normal. For example, if an image includes both a benign and a malignant finding, we classify the entire image as Malignant.

Table 5 and Table 6 present the data composition of this dataset. Because the original release of this dataset does not include a predefined partition, we created a custom split for our experiments, dividing the data into training and test sets at an approximate 9:1 ratio. Abnormal data consists of benign data and malignant data.

## 4. Results

Table 7, Table 8, Table 9, Table 10, Table 11, Table 12, Table 13, Table 14, Table 15, Table 16, Table 17 and Table 18 present the results of this study.

We presented the results of normal/abnormal classification on the VinDr dataset using five deep learning models without considering the presence of regions of interest (ROIs) in Table 7.

We presented the results of normal/abnormal classification on the VinDr dataset using five deep learning models with consideration of ROI presence in Table 8.

We presented the results of benign/malignant classification on the VinDr dataset using five deep learning models without considering ROI presence in Table 9.

We presented the results of benign/malignant classification on the VinDr dataset using five deep learning models with consideration of ROI presence in Table 10.

We presented the results of normal/abnormal classification on the CDD-CESM dataset using five deep learning models without considering ROI presence in Table 11.

We presented the results of normal/abnormal classification on the CDD-CESM dataset using five deep learning models with consideration of ROI presence in Table 12.

We presented the results of benign/malignant classification on the CDD-CESM dataset using five deep learning models without considering ROI presence in Table 13.

We presented the results of benign/malignant classification on the CDD-CESM dataset using five deep learning models with consideration of ROI presence in Table 14.

We presented the results of normal/abnormal classification on the DMID dataset using five deep learning models without considering ROI presence in Table 15.

We presented the results of normal/abnormal classification on the DMID dataset using five deep learning models with consideration of ROI presence in Table 16.

We presented the results of benign/malignant classification on the DMID dataset using five deep learning models without considering ROI presence in Table 17.

We presented the results of benign/malignant classification on the DMID dataset using five deep learning models with consideration of ROI presence in Table 18.

## 5. Discussion

This study validated our proposed methodology using datasets that provide ROI annotations. According to the evaluation metrics (accuracy, sensitivity, specificity, F1) used to compare the results of this study, the utility of the validated approach is particularly evident for large-scale datasets such as VinDr. By contrast, we observed minimal differentiation in performance metrics when applying the same method to smaller datasets such as DMID.

A primary limitation of our validated methodology is that it partitions the dataset based on ROI presence and view type, which consequently reduces the number of available training samples. We posit that this data fragmentation is a principal contributing factor to the minimal performance variance observed for the smaller dataset. Therefore, we identify data augmentation via image generation techniques as a crucially important direction for future research. This approach might simultaneously address the challenges of data scarcity and class imbalance, particularly in cases with limited data.

A key area for future work involves extension of this validation to datasets lacking such information, a task that would necessitate a preliminary ROI generation phase.

## 6. Conclusions

This study investigated the potential generalizability of a methodology that partitions mammograms based on the presence or absence of an ROI, which trains models on these subsets independently, and which subsequently integrates their inference results. We validated this approach, which is aimed at improving accuracy for both normal–abnormal and benign–malignant classification, using three public datasets and five representative deep learning models. Our findings indicate that the utility of this generalizability is particularly prominent when used with large-scale datasets such as VinDr, although it produces less discernible differences in classification performance when used with smaller datasets such as DMID.

## Figures and Tables

**Table 1:** Distribution of Normal and Abnormal Images in the VinDr Dataset.

	Normal	Abnormal	Total
train	10,724	5276	16,000
test	2682	1318	4000
total	13,406	6594	20,000

**Table 2:** Distribution of Benign and Malignant Images in the VinDr Dataset.

	Benign	Malignant	Total
train	4486	790	5276
test	1120	198	1318
total	5606	988	6594

**Table 3:** Distribution of Normal and Abnormal Images in the CDD-CESM Dataset.

	Normal	Abnormal	Total
train	305	592	897
test	36	70	106
total	341	662	1003

**Table 4:** Distribution of Benign and Malignant Images in the CDD-CESM Dataset.

	Benign	Malignant	Total
train	296	296	592
test	35	35	70
total	331	331	662

**Table 5:** Distribution of Normal and Abnormal Images in the DMID Dataset.

	Normal	Abnormal	Total
train	209	248	457
test	25	28	53
total	234	276	510

**Table 6:** Distribution of Benign and Malignant Images in the DMID Dataset.

	Benign	Malignant	Total
train	132	116	248
test	14	14	28
total	146	130	276

**Table 7:** VinDr/Normal–Abnormal/without consideration of ROI presence.

	Accuracy	Sensitivity	Specificity	F1
Resnet	0.6940	0.3042	0.8855	0.3959
EfficientNet	0.7263	0.3938	0.8896	0.4866
SwinTransformer	0.7000	0.3869	0.8538	0.4595
ConvNeXt	0.6905	0.2496	0.9072	0.3470
MobileNet	0.6755	0.2792	0.8702	0.3618

**Table 8:** VinDr/Normal–Abnormal/with consideration of ROI presence.

	Accuracy	Sensitivity	Specificity	F1
Resnet	0.7675	0.4621	0.9176	0.5670
EfficientNet	0.7448	0.5114	0.8594	0.5690
SwinTransformer	0.7458	0.5114	0.8609	0.5700
ConvNeXt	0.7128	0.5068	0.8139	0.5376
MobileNet	0.6928	0.4742	0.8001	0.5042

**Table 9:** VinDr/Benign–Malignant/without consideration of ROI presence.

	Accuracy	Sensitivity	Specificity	F1
Resnet	0.8308	0.4343	0.9009	0.4354
EfficientNet	0.8331	0.4242	0.9054	0.4330
SwinTransformer	0.8285	0.4192	0.9009	0.4235
ConvNeXt	0.8475	0.3737	0.9313	0.4241
MobileNet	0.8194	0.3636	0.9000	0.3770

**Table 10:** VinDr/Benign–Malignant/with consideration of ROI presence.

	Accuracy	Sensitivity	Specificity	F1
Resnet	0.8968	0.5202	0.9634	0.6023
EfficientNet	0.9059	0.6162	0.9571	0.6630
SwinTransformer	0.9074	0.6162	0.9589	0.6667
ConvNeXt	0.8862	0.5253	0.9500	0.5810
MobileNet	0.8945	0.58808	0.9500	0.6233

**Table 11:** CDD-CESM/Normal–Abnormal/without consideration of ROI presence.

	Accuracy	Sensitivity	Specificity	F1
Resnet	0.7547	0.7571	0.7500	0.8030
EfficientNet	0.7736	0.7000	0.9167	0.8033
SwinTransformer	0.6981	0.7429	0.6111	0.7647
ConvNeXt	0.7547	0.7143	0.8333	0.7937
MobileNet	0.6981	0.6286	0.8333	0.7333

**Table 12:** CDD-CESM/Normal–Abnormal/with consideration of ROI presence.

	Accuracy	Sensitivity	Specificity	F1
Resnet	0.9434	0.9429	0.9444	0.9565
EfficientNet	0.9245	0.9286	0.9167	0.9420
SwinTransformer	0.9528	0.9571	0.9444	0.9640
ConvNeXt	0.9340	0.9429	0.9167	0.9496
MobileNet	0.9528	0.9571	0.9444	0.9640

**Table 13:** CDD-CESM/Benign–Malignant/without consideration of ROI presence.

	Accuracy	Sensitivity	Specificity	F1
Resnet	0.6429	0.5429	0.7429	0.6032
EfficientNet	0.5143	0.3714	0.6571	0.4333
SwinTransformer	0.5571	0.4286	0.6857	0.4918
ConvNeXt	0.6000	0.6571	0.5429	0.6216
MobileNet	0.6429	0.6571	0.6286	0.6479

**Table 14:** CDD-CESM/Benign–Malignant/with consideration of ROI presence.

	Accuracy	Sensitivity	Specificity	F1
Resnet	0.6143	0.7143	0.5143	0.6494
EfficientNet	0.5857	0.6857	0.4857	0.6234
SwinTransformer	0.6429	0.6000	0.6857	0.6269
ConvNeXt	0.6286	0.5143	0.7429	0.5806
MobileNet	0.6286	0.6857	0.5714	0.6486

**Table 15:** DMID/Normal–Abnormal/without consideration of ROI presence.

	Accuracy	Sensitivity	Specificity	F1
Resnet	0.7736	0.7857	0.7600	0.7857
EfficientNet	0.6792	0.7143	0.6400	0.7018
SwinTransformer	0.7358	0.7857	0.6800	0.7586
ConvNeXt	0.8302	0.7857	0.8800	0.8302
MobileNet	0.6792	0.6786	0.6800	0.6909

**Table 16:** DMID/Normal–Abnormal/with consideration of ROI presence.

	Accuracy	Sensitivity	Specificity	F1
Resnet	0.9057	0.9643	0.8400	0.9153
EfficientNet	0.8868	0.9286	0.8400	0.8966
SwinTransformer	0.8868	0.9286	0.8400	0.8966
ConvNeXt	0.9057	0.9643	0.8400	0.9153
MobileNet	0.8679	0.8929	0.8400	0.8772

**Table 17:** DMID/Benign–Malignant/without consideration of ROI presence.

	Accuracy	Sensitivity	Specificity	F1
Resnet	0.8571	1.0000	0.7143	0.8750
EfficientNet	0.7500	0.9286	0.5714	0.7879
SwinTransformer	0.6786	0.7857	0.7857	0.7857
ConvNeXt	0.7857	0.7857	0.7857	0.7857
MobileNet	0.7143	0.8571	0.5714	0.7500

**Table 18:** DMID/Benign–Malignant/with consideration of ROI presence.

	Accuracy	Sensitivity	Specificity	F1
Resnet	0.8148	0.7692	0.8571	0.8000
EfficientNet	0.3704	0.3077	0.4286	0.3200
SwinTransformer	0.5926	0.6154	0.5714	0.5926
ConvNeXt	0.8148	0.9231	0.7143	0.8276
MobileNet	0.5926	0.5385	0.6429	0.5600

## Data Availability

All datasets used by the authors in this study are publicly available online; see references section for details.
